# Communications via the Small Leucine-rich Proteoglycans: Molecular Specificity in Inflammation and Autoimmune Diseases

**DOI:** 10.1369/0022155420930303

**Published:** 2020-07-06

**Authors:** Jinyang Zeng-Brouwers, Sony Pandey, Jonel Trebicka, Malgorzata Wygrecka, Liliana Schaefer

**Affiliations:** Pharmazentrum Frankfurt/ZAFES, Institut für Allgemeine Pharmakologie und Toxikologie, Klinikum der Johann Wolfgang Goethe-Universität Frankfurt am Main, Frankfurt am Main, Germany; Pharmazentrum Frankfurt/ZAFES, Institut für Allgemeine Pharmakologie und Toxikologie, Klinikum der Johann Wolfgang Goethe-Universität Frankfurt am Main, Frankfurt am Main, Germany; Translational Hepatology, Department of Internal Medicine I, University Clinic Frankfurt, Frankfurt, Germany; Department of Biochemistry, Faculty of Medicine, Universities of Giessen and Marburg Lung Center, Giessen, Germany; German Center for Lung Research, Giessen, Germany; Pharmazentrum Frankfurt/ZAFES, Institut für Allgemeine Pharmakologie und Toxikologie, Klinikum der Johann Wolfgang Goethe-Universität Frankfurt am Main, Frankfurt am Main, Germany

**Keywords:** autophagy, biglycan, decorin, extracellular matrix, fibromodulin, glycosaminoglycan, lumican, macrophage, proteoglycan, Toll-like receptor

## Abstract

Inflammation is a highly regulated biological response of the immune system that is triggered by assaulting pathogens or endogenous alarmins. It is now well established that some soluble extracellular matrix constituents, such as small leucine-rich proteoglycans (SLRPs), can act as danger signals and trigger aseptic inflammation by interacting with innate immune receptors. SLRP inflammatory signaling cascade goes far beyond its canonical function. By choosing specific innate immune receptors, coreceptors, and adaptor molecules, SLRPs promote a switch between pro- and anti-inflammatory signaling, thereby determining disease resolution or chronification. Moreover, by orchestrating signaling through various receptors, SLRPs fine-tune inflammation and, despite their structural homology, regulate inflammatory processes in a molecule-specific manner. Hence, the overarching theme of this review is to highlight the molecular and functional specificity of biglycan-, decorin-, lumican-, and fibromodulin-mediated signaling in inflammatory and autoimmune diseases

## Introduction

Inflammation is a tightly regulated biological response of the immune system against invading foreign objects or endogenous signals.^1,2^ Foreign objects (e.g., bacteria or viruses) express pathogen-associated molecular patterns (PAMPs) that are recognized by pattern recognition receptors to trigger an inflammatory response.^3^ The endogenous triggers of this process are called damage-associated molecular patterns (DAMPs). DAMPs originate either from inside the cell or from the extracellular matrix (ECM).^3^ It is of note that DAMPs, similar to PAMPs, are recognized by the same innate immune receptors, for example, Toll-like receptors (TLRs), RIG-I-like receptors, nucleotide-binding oligomerization domain (NOD)-like receptors, receptor for advanced glycation end products, integrins, and cluster of differentiation (CD) 44.^4^ The induction of inflammation initiated by PAMPs or DAMPs results in the release of cytokines/chemokines to protect the body against the spread of infection or uncontrolled tissue damage.^5^

The fate of inflammation, however, is determined by the resolution phase.^6^ Resolution is important to subside inflammation and is mediated through other tightly regulated mechanisms such as autophagy that is responsible for the clearance of damaged cells or cellular organelles.^7^ Chronic or uncontrolled activation of the innate immune response leads to inflammatory diseases.^8^ Similarly, chronic inflammatory response erroneously triggered against the body’s healthy tissues and activated by the adaptive immune response results in autoimmune diseases such as inflammatory bowel disease (IBD), rheumatoid arthritis (RA), multiple sclerosis (MS), systemic lupus erythematosus (SLE), and type 1 diabetes mellitus, among others.^9,10^

It is becoming increasingly clear that members of the small leucine-rich proteoglycan (SLRP) family play critical roles in both the promotion and the resolution of inflammation as canonical ECM-derived DAMPs.^11–14^ The SLRPs are a family of proteoglycans that are major components in the ECM with common leucine-rich repeat (LRR) region in their core protein.^15,16^ The SLRP family has been expanded to five classes based on homologies at the genomic and protein level.^17^ The class I SLRPs, decorin and biglycan, as well as lumican and fibromodulin that belong to class II, are the best characterized members of the SLRP family.^18^ SLRPs are present in various tissues in either an ECM-bound or soluble form and have important structural and signaling functions.^16,17,19–25^ As signaling molecules, SLRPs regulate both pathogen-mediated and sterile inflammation during innate and adaptive immune responses.^3,26^ These interactions are tightly coordinated and mediated through specific receptors, coreceptors, adaptor molecules, and specific SLRP regions.^14,16,22–24^

It becomes obvious that besides their structural homology, SLRPs regulate inflammatory processes in a molecule-specific manner. In this review, we aim to discuss recent mechanisms of biglycan-, decorin-, lumican-, and fibromodulin-mediated aggravation and resolution of inflammation. The functional specificity of SLRP signaling in inflammatory and autoimmune diseases will be emphasized.

## Biglycan Signaling in Inflammatory and Autoimmune Diseases

### The ECM-bound and Soluble Form of Biglycan

Biglycan, a member of class I SLRPs, consists of a 42-kDa protein core containing 10 LRRs that are covalently bound to one or two chondroitin/dermatan sulfate glycosaminoglycan (GAG) side chains.^16,21^ Through its protein core and GAG chains, biglycan interacts with various ECM components, for example, collagen types I, II, III, IV, and VI and elastin, thereby playing a crucial structural role in majority of tissues.^27–31^

It is now well accepted that biglycan exists in the blood and organs in two forms: the physiological form that is ECM-sequestered and the soluble form that is associated with tissue stress and injury.^11,32–34^ Soluble biglycan is generated via the proteolytic release of ECM-bound biglycan.^35^ This is the fastest mechanism to protect tissues with both full-length and fragmented biglycan during an emergency. This is followed by de novo expression and secretion of full-length biglycan by macrophages and later on by tissue-resident cells.^11,35^ Both ECM-bound and soluble biglycan can influence multiple signaling pathways by interacting with various growth factors and cytokines, for example, transforming growth factor beta (TGF-β); tumor necrosis factor-α (TNF-α); bone morphogenetic protein (BMP)-2, -4, -6; and Wnt-1-induced secreted protein 1 (WISP1).^36–39^ In contrast, only soluble form of biglycan can interact with and signal through TLR2/TLR4. Although biglycan binds to TLRs at the protein core,^40^ the GAG side chains are required for its signaling via TLR2 and TLR4.^11,35^ All studies to date show that only intact biglycan containing both protein core and GAG side chains is capable of triggering pro-inflammatory signaling.^11,32,41,42^

There are several reviews that address the complexity of biglycan signaling in detail.^3,4,14,19–23,25,33,35,43–48^ In this article, we will briefly summarize the interaction of biglycan with TLR2/TLR4 and the decisive role of TLR coreceptors and adaptor molecules in regulating the downstream outcomes of the nuclear factor kappa-light-chain-enhancer of activated B-cells (NF-κB) and inflammasome signaling pathways. We will emphasize the role of biglycan in bridging innate and adaptive immune responses. Finally, we will summarize current knowledge regarding the input of biglycan in inflammatory and autoimmune diseases.

### Biglycan Acts as a Danger Signal Through TLR2 and TLR4

**Figure 1.**
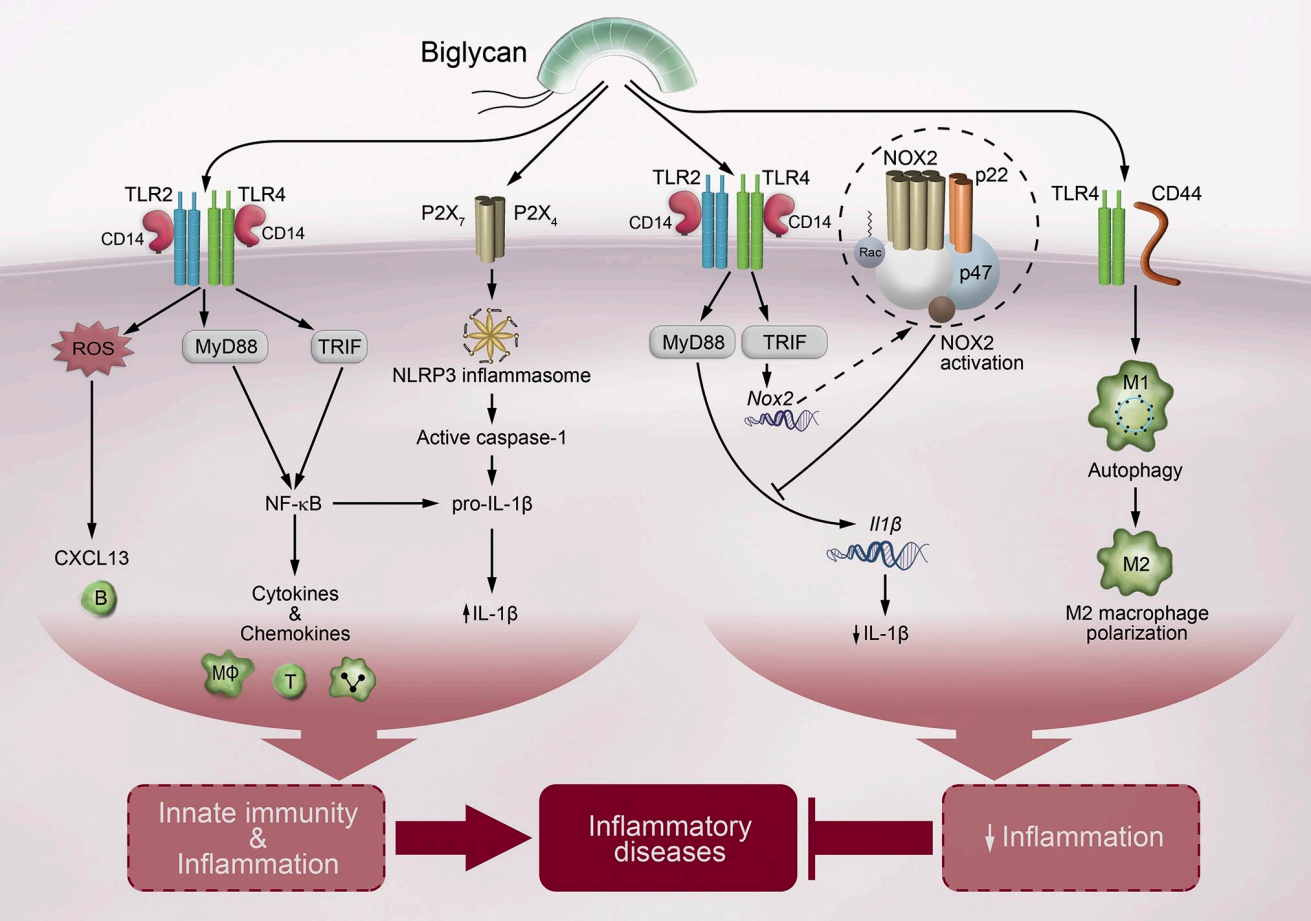
Biglycan determines pro- and anti-inflammatory signaling response by switching between TLR2/TLR4/CD14 and TLR4/CD44. Soluble biglycan via TLR2/TLR4/CD14 activates the pro-inflammatory NF-κB signaling, leading to chemokine and cytokine production, immune cell recruitment, and pro-IL-1β production. Based on the same signaling, biglycan induces ROS generation and production of the B-cell chemoattractant CXCL13. In addition, biglycan clusters the purinergic receptors P2X_4_/P2X_7_ to trigger the NLRP3 inflammasome assembly, subsequently leading to the turnover of pro-IL-1β, by activated caspase-1, to active IL-1β. Together, these responses facilitate innate immunity and inflammation, promoting inflammatory and autoimmune diseases. However, soluble biglycan can also exert anti-inflammatory signals. Biglycan induces the expression of NOX2 via the TLR2/TLR4/TRIF pathway, which ultimately leads to the inhibition of biglycan-TLR2/TLR4/MyD88-mediated IL-1β production. Furthermore, biglycan decreases inflammation by induction of autophagy. Through TLR4 and its coreceptor CD44, biglycan induces autophagy of M1 macrophages, thereby elevating the number of anti-inflammatory M2 macrophages. These responses can thereby inhibit unmitigated inflammation during inflammatory and autoimmune diseases. Abbreviations: CD, cluster of differentiation; CXCL, chemokine (C-X-C) motif ligand; IL, interleukin; NF-κB, nuclear factor kappa-light-chain-enhancer of activated B-cells; MyD88, myeloid differentiation primary response 88; NLRP3, nucleotide-binding oligomerization domain, leucine-rich repeat and pyrin domain containing 3; NOX, NADPH oxidase; ROS, reactive oxygen species; TLR, Toll-like receptor; TRIF, TIR domain-containing adaptor-inducing interferon-β.

Research over the last 15 years provides concrete evidence that soluble biglycan acts as ECM-derived danger signal in macrophages.^3,11^ Biglycan binds to TLR2 and TLR4 with an affinity comparable to respective pathogen-derived ligands of TLR2/TLR4, thereby mimicking the response of Gram-positive and Gram-negative bacteria.^11,40,41^ Downstream of both receptors, biglycan triggers NF-κB-, p38-, and extracellular signal-regulated kinase (ERK) signaling.^11^ This leads to the activation of various inflammatory cytokines, for example, TNF-α, macrophage inflammatory protein 2, and interleukin (IL)-1β, as well as chemokines, for example, C-C motif chemokine ligand (CCL) 2, CCL5, C-X-C motif ligand (CXCL) 1, and CXCL13 (Fig. 1).^11,32,49^

Furthermore, by clustering TLR2/TLR4 with the P2X_4_/P2X_7_ purinergic receptors, biglycan autonomously triggers the nucleotide-binding oligomerization domain, leucine-rich repeat and pyrin domain containing (NLRP) 3 inflammasome, thereby activating caspase-1 and inducing the maturation and secretion of IL-1β (Fig. 1).^41^

### Biglycan Regulates Signaling Outcome by Selectively Interacting With TLRs, Their Adaptor Molecules, and Coreceptors

The initial finding that biglycan utilizes both TLRs to trigger “sterile” inflammation was verified by careful analysis of biglycan-mediated recruitment of neutrophils, macrophages, and T-cells into the kidney.^40^ It became obvious that biglycan, by “choosing” one of the TLRs or their specific adaptor molecules, the myeloid differentiation primary response 88 (MyD88) or Toll/IL-1R domain-containing adaptor-inducing interferon (IFN)-β (TRIF), triggers specific downstream signaling outcome (Fig. 1).^40^ Accordingly, by using the TLR2/TLR4/MyD88 pathway, biglycan activates the chemoattractants CXCL1, CXCL2, and CCL2 to recruit neutrophils and macrophages.^40^ In contrast, infiltration of T-cells is triggered by biglycan via the TLR4/TRIF pathway and production of CCL5 and CXCL10.^13,40^ Selective signaling of biglycan via TLR2 or TLR4 and their adaptor molecules is even more complex in terms of the T-helper (Th) 1 and Th17 cell recruitment.^13^ Through TLR4/TRIF, biglycan stimulates infiltration of CXCR3-positive Th1 and Th17 cells. However, CC chemokine receptor 6–positive Th17 cells are recruited by biglycan via TLR2 and TLR4 and their common adaptor MyD88.^13^

Furthermore, biglycan initiates a crosstalk between TLR and sphingosine kinase (SphK) 1 signaling or reactive oxygen species (ROS) signaling, resulting in various downstream outcomes.^50,51^ Accordingly, biglycan stimulates the production and activation of SphK1 in a TLR4/TRIF-dependent manner. Of particular note is the biglycan-triggered expression of the B-cell chemoattractant CXCL13 in peritoneal macrophages and splenic dendritic cells that is mediated by TLR2 and TLR4 and involves ROS as part of their signaling cascade (Fig. 1).^32^ For further details, please refer to recent reviews.^13,25,52^

It is of note that biglycan, besides acting as a canonical DAMP, exerts additional anti-inflammatory effects. Up to now, two mechanisms of biglycan-mediated inhibition of the inflammatory response are described.^34,50^ Biglycan is involved in TLR4/TRIF-dependent production of NADPH oxidase (NOX) 2 (Fig. 1).^50^ Furthermore, biglycan triggers the translocation of NOX2 from the cytoplasm to the plasma membrane, resulting in the formation and activation of the NOX2 complex. Active NOX2 inhibits biglycan/TLR2/TLR4/MyD88-dependent IL-1β production, thereby reducing inflammation (Fig. 1).^24,50^ It is tempting to speculate that this mechanism is involved under physiological conditions to avoid the pro-inflammatory effects of biglycan released from the ECM.

Recent studies have provided a new milestone in our understanding of how biglycan influences the outcome of inflammatory diseases. Biglycan promotes a switch between inflammation and autophagy via selectively choosing CD14, the coreceptor of TLR2/TLR4, or CD44, the TLR4 coreceptor.^14,34^ By interacting with either TLR2/CD14 or TLR4/CD14, biglycan acts as a canonical DAMP, thereby promoting recruitment of pro-inflammatory M1 macrophages into the kidney.^34,52^ In contrast, binding of biglycan to the TLR4 coreceptor, CD44, causes M1 macrophage autophagy (Fig. 1).^34^ This is associated with enhanced number of alternatively polarized anti-inflammatory M2 macrophages and reduced tissue damage (Fig. 1).^34^ Thus, biglycan, by selecting a respective coreceptor for TLRs, promotes either inflammation or autophagy, thereby determining disease chronification or resolution.

### Biglycan in Inflammatory Diseases

There is a plethora of reports underscoring the mechanisms of biglycan-dependent regulation of inflammation under in vivo conditions.^25,32,53^ In this review, the most striking examples will be addressed. For further details, please refer to recent reviews on biglycan.^3,4,14,19–23,25,33,35,43–48^

The importance of biglycan signaling in pathogen-dependent inflammation is clearly demonstrated in a mouse model of lipopolysaccharide (LPS)-induced sepsis as biglycan-deficient mice markedly displayed prolonged survival time associated with lower plasma levels of the two major inflammatory cytokines TNF-α and IL-1β.^11,41^

There are several examples for biglycan self-directed sterile inflammation in vivo. The critical role of biglycan in the activation of NLRP3 inflammasome is confirmed in experimental models of renal inflammation and fibrosis.^32,41^ In lupus nephritis (LN) and unilateral ureteral obstruction, biglycan deficiency causes lower levels of active caspase-1 and mature IL-1β, which is associated with a reduction in renal tissue damage.^41,49^ In contrast, overexpression of soluble biglycan aggravates kidney damage in LN and ischemia reperfusion injury (IRI).^32,49^

Furthermore, in biglycan-deficient and biglycan-overexpressing mice challenged by renal IRI, the significance of biglycan-dependent regulation of SphK1 and NOX2 in the kidney is clearly demonstrated.^51,54^ Also, there are several reports demonstrating how biglycan orchestrates inflammatory signaling in cancer development.^23–26^

Taken together, there is growing evidence for a critical role of biglycan in various inflammatory diseases. It is becoming apparent that soluble biglycan triggers sterile inflammation autonomously. In pathogen-mediated diseases, biglycan potentiates the inflammatory response via a second TLR that is not involved in pathogenic sensing, for example, via TLR2 in LPS-mediated sepsis.

### Biglycan in Autoimmune Diseases

Elevated soluble biglycan levels are reported in several autoimmune diseases, for example, RA, autoimmune perimyocarditis, diabetes mellitus type 1, and LN.^13,42,55^ In LN, soluble biglycan triggers innate and adaptive immune responses, thereby controlling the progression and outcome of this disease.^13,32^ In MRL/lpr mice lacking or overexpressing soluble biglycan, a critical role of this proteoglycan for CXCL13-dependent recruitment of B1- and B-lymphocytes is proven.^32^ Furthermore, biglycan in LN triggers the production of various chemoattractants for neutrophils, macrophages, and T-cells, thereby regulating albuminuria and degree of kidney damage.^32^ Importantly, elevated plasma levels of biglycan in correlation with albuminuria and disease progression were detected in patients suffering from LN.^32^

Furthermore, biglycan is an important trigger of CXCL9/CXCL10-mediated recruitment of Th1 and Th17 cells in LN.^13^ In LN patients and MRL/lpr mice, increased plasma concentration of soluble biglycan correlates with enhanced CXCL9 and CXCL10 levels.^13^ In addition, by interacting with TLR2/TLR4 receptors and their protein adaptor molecules MyD88 and TRIF, biglycan influences major histocompatibility complex (MHC) I– and MHC II–restricted T‑cell cross-priming.^53^ In a model of experimental autoimmune perimyocarditis, biglycan–TLR4 interaction induces cardiomyocyte antigen presentation to prime T-cells.^53^

Biglycan is also involved in the pathogenesis of diseases which involve dysregulated ECM remodeling, for example, RA.^56–58^ Increased levels of soluble biglycan and anti-biglycan antibodies were detected in the synovial fluid of patients suffering from RA.^56,57^ In addition, it has been reported that anti-biglycan antibody caused collagen fiber decomposition.^56,57^ Biglycan was therefore proposed as an initiator of tissue destruction in RA.^56,57^ Moreover, in a rat model of collagen-induced RA, fragments of biglycan generated by matrix metalloproteinase (MMP) degradation positively correlated with the progression of RA.^58^

Up to now, inflammatory signaling of biglycan and its relevance under disease condition is the best characterized among all SLRPs. Thus, biglycan tightly regulates inflammation, and thereby inflammatory diseases, by orchestrating signaling in the direction of either resolution or chronification, in a molecule-specific way.

## Decorin-dependent Regulation of Inflammation

### Structural and Functional Characteristics of Decorin

Decorin is another class I SLRP that is structurally close to biglycan, sharing 55% homology with it.^59^ It is composed of a 40-kDa protein core containing 10 LRRs and a single chondroitin/dermatan sulfate GAG side chain attached to its N-terminal site.^60^ Decorin is mostly found in the ECM matrix of various types of connective tissues such as skin and bone,^33^ where it interacts with collagen I exerting its ability for collagen fibrillogenesis.^61–65^ Besides its structural role, decorin is also one of the most versatile SLRPs that regulates a vast range of cellular processes, including angiogenesis,^66,67^ myocardial infarction,^68^ innate immunity,^22^ fibrosis,^69^ wound healing,^70^ tumor growth and autophagy.^71–75^ This functional diversity arises from a broad array of interactions between decorin and its various binding partners that encompass ECM constituents, cellular receptors, growth factors, proteases/enzymes, and other signaling molecules.^71,76,77^ The majority of decorin interactions with its binding partners occurs via the specific binding motifs in its protein core, whereas some interactions can also involve its GAG chain.^62,78^ The complexity of decorin interacting networks and the biological functions of these multifaceted interactions have previously been addressed in detail in several reviews.^71,76,77,79–82^

### Decorin Triggers Pro-inflammatory Effect in Macrophages

**Figure 2.**
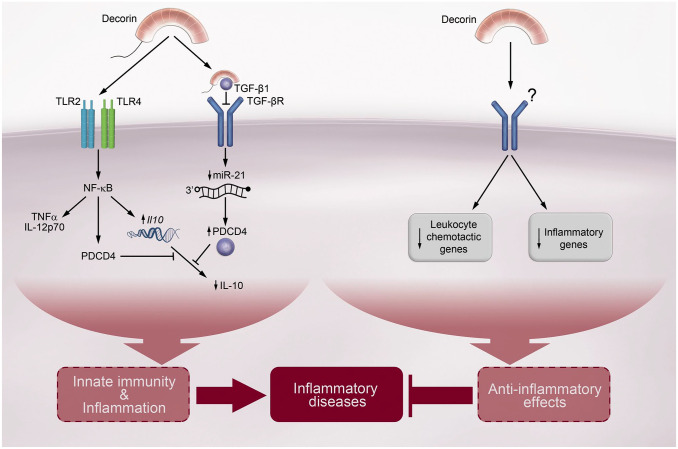
Decorin protein structure is critical in determining its pro- and anti-inflammatory signaling response. The proteoglycan form of decorin, comprising the protein core and GAG chain, promotes innate immunity and inflammation by dual mechanisms. On one hand, decorin, by binding to TLR2/TLR 4, activates NF-κB signaling and induces the expression of pro-inflammatory cytokines *Tnfα, Il-12p70*, and *Pdcd4*, as well as the anti-inflammatory cytokine *Il10*. On the other hand, by binding to TGF-β1, decorin blocks TGF-β1 binding and its subsequent activation of the TGF-β receptor (TGFβR), thus inhibiting the maturation of microRNA-21, a posttranscriptional inhibitor of PDCD4. Increased PDCD4 abundance reduces levels of IL-10. This results in inflammation, which in chronic conditions can lead to inflammatory and autoimmune diseases. In contrast, the decorin protein core promotes anti-inflammatory effects by suppressing the expression of leukocyte chemotactic genes and inflammatory genes, albeit the exact receptors involved in the signaling are still unknown. Nevertheless, their anti-inflammatory effect has important functions in mitigating inflammation during inflammatory and autoimmune diseases. Abbreviations: GAG, glycosaminoglycan; IL, interleukin; NF-κB, nuclear factor kappa-light-chain-enhancer of activated B-cells; TLR, Toll-like receptor; TNF, tumor necrosis factor; PDCD4, programmed cell death protein 4; TGF, transforming growth factor.

Soluble decorin, similar to biglycan, is ascertained as an endogenous ligand of TLR2 and TLR4, acting as a canonical DAMP and regulator of pathogen-mediated and sterile inflammation (Fig. 2).^12^ Akin to biglycan, only intact decorin encompassing both protein core and GAG chain can trigger a pro-inflammatory response in macrophages.^12,43^

Binding of decorin to TLR2 and TLR4 in macrophages results in the rapid activation of p38, ERK1/2, and NF-κB pathways and enhances the synthesis of pro-inflammatory cytokines TNF-α and IL-12p70 (Fig. 2).^12^ Furthermore, by signaling through TLR2/TLR4, decorin acts as a transcriptional inducer of tumor suppressor programmed cell death 4 (PDCD4), a unique regulator of both tumorigenesis and inflammation (Fig. 2).^12,83^ In addition, by a reduction in mature microRNA (miR)-21, an oncogene and a posttranscriptional repressor of PDCD4, decorin further contributes to the enhancement of PDCD4 protein abundance (Fig. 2).^12^ This occurs independent of TLR2/TLR4 and is based on decorin-mediated inactivation of TGF-β1, which normally enhances the levels of precursor and mature miR-21.^12,84^ The subsequent increase in PDCD4, a specific translational suppressor of IL-10, finally results in lower anti-inflammatory IL-10 protein levels (Fig. 2).^12^

Taken together, decorin creates a pro-inflammatory environment by the stimulation of pro-inflammatory PDCD4, TNF-α, and IL-12, as well as by the inhibition of immunosuppressive TGF-β1 and anti-inflammatory IL-10 (Fig. 2). Hence, this pro-inflammatory pathway is evoked in a decorin-specific manner that differs from biglycan signaling.

### Decorin in Inflammatory Diseases

Decorin-driven inflammatory signaling was verified in vivo in sepsis and tumor xenografts.^12^ In LPS-induced septic mice, high levels of decorin mRNA and protein are detected in septic lungs and macrophages.^12^ In contrast, decorin deficiency in septic mice leads to reduced PDCD4 abundance and enhanced expression of miR-21 and IL-10, which are associated with attenuated pro-inflammatory responses. This study was corroborated by a subsequent finding that LPS promoted PDCD4 degradation and IL-10 production in macrophages.^85^

In a model of tumor xenograft growth, adenovirus-mediated overexpression of decorin causes TLR2/TLR4-driven synthesis of PDCD4, TNF-α and IL-12, and TGF-β1/miR-21-mediated inhibition of PDCD4 suppression.^12^ In consequence, the immune reaction is shifted to a more apoptotic and inflammatory response with strong anti-tumorigenic effects, resulting in a marked retardation of tumor growth.^12^ This, along with the enhancement of the tumor suppressor PDCD4 and the reduction of the oncogene miR-21, might represent an attractive approach for cancer therapy.

There are no doubts about the pivotal role of decorin as an inhibitor of tumor growth and metastasis.^77^ This is based on the ability of decorin to engage multiple receptor tyrosine kinases and to act as a signaling molecule regulating angiogenesis.^86^ Even though the relationship between inflammation, immunity, and cancer is well established,^87^ studies addressing decorin-dependent regulation of tumor inflammation are still required. Further details regarding oncosuppressive functions of decorin are included in recent thematic reviews.^23,76,79,88–90^

The pro-inflammatory role of decorin is further underscored by findings demonstrating that overexpression of pancreatic decorin is associated with prolonged inflammation in chronic pancreatitis.^91^ This is due to decorin-dependent overexpression of the chemoattractant CCL2, resulting in enhanced recruitment of mononuclear cells to the injury site and maintenance of inflammation.^91^

Maintenance of inflammation through decorin-mediated pro-inflammatory signaling was also observed in delayed-type hypersensitivity (DTH).^92,93^ In an oxazolone-mediated mouse model of contact dermatitis, decorin deficiency decreased DTH progression based on the reduced expression of inflammatory cytokines, defects in CD8^+^ leukocyte recruitment, and altered functions of IFN-γ.^92,93^ Furthermore, in a murine model of allergic asthma, lack of decorin resulted in an abolished pulmonary inflammation and increased expression of anti-inflammatory *Il10* and *Foxp3* in CD4^+^CD25^+^ T-cells, causing reduction in lung tissue damage.^94,95^

Hence, the majority of reports addressing the role of decorin in inflammation clearly stress pro-inflammatory effects mediated by this SLRP. Remarkably, analysis of the global gene expression profile of the tumor microenvironment in a triple-negative orthotopic breast carcinoma xenograft model revealed that the leukocyte chemotactic and inflammatory genes are the most significantly downregulated by decorin protein core (Fig. 2).^96^ It is of note that these findings are not contrary to other reports identifying decorin as a pro-inflammatory SLRP. It is known that decorin binds to TLR2/TLR4 via its protein core.^12^ However, an intact decorin, consisting of the protein core and one GAG side chain, is required for TLR2/TLR4-mediated signaling.^12^ Therefore, it is tempting to speculate that decorin protein core acts as a non-signaling TLR2/TLR4 agonist and inhibits binding of other DAMPs from the tumor microenvironment to TLR2 and TLR4, thereby inhibiting inflammation. Future studies are required to further clarify signaling mechanisms of the decorin-mediated inflammatory response. It is of particular interest to elucidate whether decorin triggers inflammation only through TLR2/TLR4 and TGF-β1 or whether additional signaling through several receptor tyrosine kinases is involved in this process.

### Decorin in Autoimmune Diseases

There are several reports suggesting the involvement of decorin in the progression of autoimmune diseases.^97,98^ A recent study identified decorin as a crucial trigger of sterile inflammation in an NOD.B10 mouse model of primary Sjögren’s syndrome (pSS),^97^ a chronic autoimmune disease characterized by exocrine gland dysfunction and immune hyperactivity.^99^ Mechanistically, decorin via TLR4 signaling stimulates the production of TNF-α and several other inflammatory cytokines in splenocytes.^98^ Surprisingly, the inflammatory cytokine profile evoked by decorin/TLR4 differs from that induced by LPS/TLR4.^98^

There are several explanations for this distinct signaling outcome. As pharmacological inhibitors and neutralizing antibodies were used in these studies to identify the TLR conveying the decorin signals, a potential interaction of TLR2 is not completely excluded.^12^ Furthermore, decorin-mediated crosstalk between TLR4 and TGF-β1 signaling should be considered.^12^ This is conceivable because an enhanced proteolytic cleavage of decorin correlated with elevated TGF-β levels in saliva and exocrine glands from the NOD pSS mice.^100^ Moreover, multiple interactions of decorin with receptor tyrosine kinases may provide another level of complexity into the inflammatory signaling of decorin.^86^

In contrast to pSS where decorin acts as an inducer of the disease phenotype,^97,98^ in experimental IBD, decorin has protective effects on intestinal cells.^101^ IBD is an autoimmune disease characterized by chronic inflammatory gastrointestinal disorders.^101^ The pathogenesis of IBD is a complex process that involves dysregulation of both inflammation and autophagy.^102^ Decorin is a well-known inducer of inflammation and autophagy.^89^ Indeed, in the intestinal tissues of IBD mouse, enhanced decorin expression was associated with increased number of autophagosomes and elevated levels of autophagy-associated proteins.^101^ The reason why decorin promotes either inflammation or autophagy in autoimmune diseases is still a matter of debate. It is tempting to speculate that decorin, similar to biglycan,^34^ by choosing the coreceptor for TLR4, is switching the signaling pathway from inflammation to autophagy. It is also possible that the expression level of inflammatory and autophagic receptors for decorin in various tissues determines which signaling will be conveyed by decorin. Thus, it is increasingly evident that decorin-dependent signaling crosstalk between inflammation and autophagy should be addressed in more detail.

## Lumican-specific Regulation of the Inflammatory Response

### The Role of Lumican Under Physiological Conditions

Lumican is a 40-kDa proteoglycan that belongs to the class II subfamily of SLRPs and was initially described as one of the major keratan sulfate proteoglycans in the adult cornea.^103–106^ Besides the cornea, high level of lumican has been found in various types of tissues, including artery, aorta, dermis, lung, kidney, and intervertebral discs.^104,107,108^ However, in these organs, lumican is present as a glycoprotein in contrast to the cornea where it is present as a keratan sulfate proteoglycan.^104^ Lumican regulates collagen assembly in the cornea and plays a crucial role in cell migration and proliferation during embryonic development and tissue repair.^104,109–112^ Apart from its physiological role as a structural component of the ECM, lumican is also involved in the regulation of cell functions such as growth, apoptosis, migration, invasion, and angiogenesis.^113,114^ For more details, please refer to recent review papers on the structural and biological functions of lumican.^15,16,115–124^

### Mechanisms of Lumican-dependent Regulation of Inflammation

**Figure 3.**
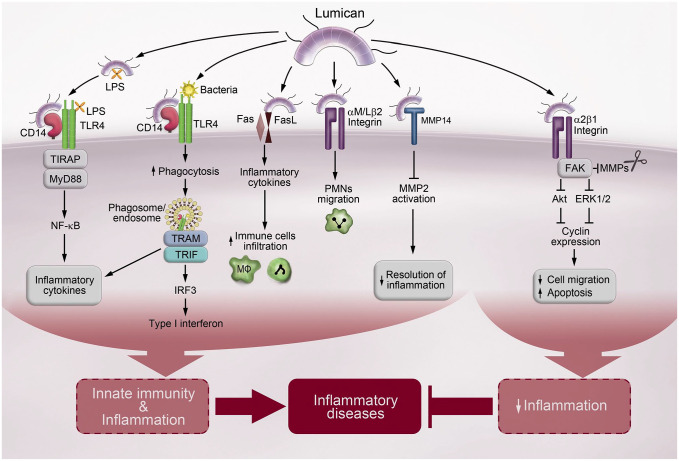
Lumican modulates innate immunity and inflammation via multiple pathways and influences the outcomes of inflammatory and autoimmune diseases. In pathogen-mediated inflammation, lumican forms a complex with the LPS and through interaction with CD14 presents it to the TLR4, thereby triggering TIRAP/MyD88-mediated signaling that causes NF-κB activation and increased expression of inflammatory cytokines. Lumican also interacts with bacteria in a TLR4/CD14-dependent manner. Consequently, the TLR4–CD14–bacterial complex activates phagocytosis and is internalized into the endosomes. Endosomal TLR4 interacts with adaptor molecules, TRAM and TRIF, to activate IRF3, which leads to type I interferon production. The endosomal TRAM–TRIF adaptor complex, independent of IRF3, also leads to the production of inflammatory cytokines. Inflammatory cytokines are also produced by lumican binding to the Fas–FasL complex, which increases infiltration of neutrophils and macrophages. Similarly, by binding to integrin subunits β_2_, α_M_, and α_L_, lumican promotes PMN cell migration, which also contributes to innate immunity, inflammation, and inflammatory diseases. Lumican via interaction with MMP14 blocks the activation of MMP2 and suppresses resolution of inflammation during inflammatory diseases. In contrast, interaction of lumican with α2β1 integrin modifies FAK signaling, which inhibits MMP bioactivity and Akt and ERK1/2 downstream signaling. As Akt and ERK1/2 are involved in cyclin expression, lumican-mediated inhibition of these pathways leads to decreased cell migration and increased apoptosis, and thereby a reduction in inflammation which can have protective effects during inflammatory and autoimmune diseases. Abbreviations: CD, cluster of differentiation; ERK, extracellular signal-regulated kinase; FAK, focal adhesion kinase; FasL, Fas ligand; IRF3, interferon regulatory factor 3; LPS, lipopolysaccharide; MMP, matrix metalloproteinase; MyD88, myeloid differentiation primary response 88; TIRAP, adaptor molecule associated with Toll-like receptors; TLR, Toll-like receptor; TRIF, TIR-domain-containing adaptor-inducing interferon-β; TRAM, TRIF-related adaptor molecule; PMN, polymorphonuclear.

An increasing number of reports have asserted that besides its physiological functions, lumican is also involved in the regulation of innate immunity.^111,125–128^ However, in contrast to biglycan^11^ and decorin,^43^ lumican does not act as a DAMP but instead promotes pathogen-dependent signaling. The lumican core protein forms a complex with bacterial LPS component and binds to CD14, the TLR4 coreceptor, on the surface of macrophages and neutrophils, thereby presenting the LPS–CD14 complex to TLR4 (Fig. 3).^126^ TLR4 activated by LPS–CD14 complex triggers the synthesis of inflammatory cytokines via its adaptor molecules, TIRAP and MyD88, and NF-κB.^126,129^ Accordingly, in LPS-induced septic mouse model, lumican-deficient mice are hyporesponsive to LPS infection, exerting reduced serum levels of pro-inflammatory TNF-α, IL-1β, and IL-6 cytokines.^130^ Furthermore, in mice infected with *Pseudomonas aeruginosa*, lumican binds to the bacteria and CD14 and presents the complex to TLR4, thereby driving bacterial phagocytosis (Fig. 3).^131^ Internalized TLR4–CD14–bacterial complex through adaptor molecules, TRIF and TRIF-related adaptor molecule (TRAM), triggers signals activating the interferon regulatory transcription factor (IRF) 3, thereby stimulating type I interferon production. In parallel, TRAM–TRIF complex promotes the secretion of pro-inflammatory cytokines (Fig. 3).^132^ Taken together, these studies uncover a molecule-specific role of lumican in promoting TLR4- and CD14-dependent pathogen sensing.^3,126^

Besides its effect on TLR4-mediated pathogen recognition, lumican modulates inflammatory response by regulating Fas ligand (FasL)–Fas signaling (Fig. 3).^111^ Binding of FasL to the surface of monocytes and macrophages induces pro-inflammatory cytokine production.^133^ It has been shown in vitro and in a mouse model of corneal inflammation that lumican binds to FasL and facilities induction of Fas signaling. These triggers enhanced inflammatory cytokine production and recruitment of neutrophils and macrophages (Fig. 3). Accordingly, corneal injury in lumican-null mice caused lower Fas protein abundance, reduced Fas–FasL signaling, and decreased the number of infiltrating neutrophils and macrophages, followed by dampened cytokine production and delayed healing.^111,134^

Another mechanism of lumican-mediated regulation of the inflammatory response is related to its interaction with MAC-1 (αM/β2) and LFA-1 (αL/β2),^125^ the two major cell surface integrins of polymorphonuclear (PMN) leukocytes (Fig. 3).^125^ By binding to both integrins, lumican promotes PMN leukocyte migration.^125^ PMN leukocytes are crucial regulators in inflammatory and autoimmune diseases.^134^ PMN trafficking toward the sites of inflammation is an initial phase of inflammatory diseases.^127^

The directional migration of PMNs through the ECM is a complex multistep process that involves several α- and β-integrin interactions with ECM proteins.^127^ There is strong evidence that lumican interacts with the β_2_, α_M_, and α_L_ integrin subunits.^128^ It is of note that lumican was detected on the surface of peritoneal PMNs, but not on blood and bone marrow PMNs, suggesting that PMNs acquire lumican after they exit circulation.^128^ This suggests that lumican might be involved in PMN extravasation. Indeed, in vivo lumican has a stimulatory role in the process of PMN extravasation during the early inflammatory phase of mouse corneal epithelium healing.^125^

Recent reports provide evidence for a direct interaction between lumican and MMP14 (Fig. 3).^135–137^ Lumican binds to the catalytic domain of MMP14 with an affinity of K_D_ ~275 nM and competitively inhibits MMP14 activity.^135^ Furthermore, lumican downregulates the MMP14 expression in endothelial and mesenchymal stem cells.^138,139^ There are several hints that MMP14 interferes with the regulation of inflammatory response.^136^ It has been shown that MMP14 deficiency enhances pulmonary inflammation and increases mortality in neonatal endotoxemia.^136^ This is associated with impaired MMP2 activation and enhanced DAMP accumulation in the lungs.^136^ Therefore, it is conceivable that lumican-dependent inhibition of the MMP14 activity decreases resolution of inflammation (Fig. 3).

### Lumican Plays Regulatory Roles in Resolution of Inflammation

Apart from its pro-inflammatory effects, lumican might also have a potential role in the modulation of cell migration and adhesion during tissue inflammation and repair via binding to α2β1 integrin and TGF-β receptor (TGFβR).^140,141^ It is reported that in diffuse intrinsic pontine glioma cells, lumican core protein can inhibit cell migration via direct interaction with α2β1 integrin (Fig. 3).^140^ Through this binding, lumican restricts the focal adhesion kinase signaling, resulting in the inhibition of (1) MMP activity, (2) ERK1/2 signaling pathway, and (3) Akt signaling pathway (Fig. 3).^140^ Inhibition of ERK1/2 and Akt downstream signaling pathways reduces cell motility and induces apoptosis.^142^ Based on these observations, it is tempting to speculate that lumican plays an anti-inflammatory role through blockage of ERK1/2 and Akt pathways in inflammatory cells (Fig. 3).

Furthermore, lumican regulates adhesion of osteosarcoma cells by modulating TGF-β2/Smad2 signaling pathway.^141^ Although the exact mechanisms of lumican inhibition of TGF-β2 signaling are still unclear, it is known that lumican directly binds to TGFβR1 (ALK5) and promotes epithelium wound healing.^143^ The consequences of lumican–TGFβR1 complex formation on the binding of TGF-β to TGFβR and TGF-β downstream signaling require further investigations. As TGF-β signaling is involved directly and indirectly in almost each regulatory step of immunity and inflammation,^144^ it is predictable that various effects of lumican on the inflammatory response will be reported in the future.

### Lumican in Inflammatory and Autoimmune Diseases

In light of the great potential of lumican to be involved in the pathogenesis of autoimmune diseases, the scarcity of data in this field is surprising. It has been reported that lumican is overexpressed in ulcerative colitis induced by trinitrobenzene sulfonic acid (TNBS) in mice and regulates the early stage of inflammation in the colon.^145^ In this model, the wild-type mice revealed an increased activation of NF-κB, which was associated with enhanced levels of CXCL1, TNF-α, and higher number of infiltrating neutrophils in the colon.^145^ In contrast, the TNBS-treated lumican-null mice displayed markedly reduced inflammatory response, which was associated with enhanced ulceration and necrosis in the colon.^145^ Overall, this study indicates a key role for lumican in maintaining intestinal homeostasis by regulating the inflammatory response and protecting tissue damage in ulcerative colitis.

Furthermore, lumican regulates the progression of MS,^146^ a chronic autoimmune disease of the central nervous system.^147^ Accordingly, lumican-deficient mice displayed an earlier onset and enhanced disease severity in experimental autoimmune encephalomyelitis (EAE).^146^ Several studies have implicated that Th17 cells play an essential role in the development of both human MS and animal model EAE.^148–150^ Mechanistically, lumican promotes apoptosis of Th17 cells via the Fas–FasL signaling pathway and inhibits the expression of pro-inflammatory IL-17, a Th17 cytokine.^146^ Thus, lumican acts as an endogenous inhibitor of Th17 cells, negatively regulating Th17 cell–mediated inflammation in MS.

Hence, lumican- and biglycan-dependent effects on Th17 cells in autoimmune diseases accentuate the major message of this review that SLRPs, in a molecule-specific manner, tightly regulate inflammation. While lumican in MS is decreasing the number of Th17 cells through their apoptotic death,^146^ biglycan via TLR2/TLR4 is promoting recruitment of Th17 cells in LN.^13^

## Fibromodulin Regulates Inflammation by Interfering With the Complement and TGF-β1 Signaling Pathways

### The Role of Fibromodulin in Tissue Homeostasis

Fibromodulin, a class II SLRP, is characterized by a 42-kDa protein core attached covalently to one or more keratan sulfate chains, with the entire size of the glycanated form measuring up to 82 kDa.^151^ Fibromodulin, initially described as a cartilage proteoglycan,^152^ is ubiquitously present in the ECM of connective tissues where it plays a central role in the organization of collagen fibrils.^153^ By interacting with lysyl oxidase, a collagen crosslinking enzyme, fibromodulin regulates the ECM composition to provide an environment favorable for cellular turnover.^154^ Similar to biglycan and decorin, fibromodulin regulates TGF-β1 signaling by sequestering the active form of this growth factor in the ECM.^155^ In addition, fibromodulin exerts various tissue-specific effects. It plays a critical role in muscle development by controlling myogenic factors and myostatin. It also promotes vasculature development and regeneration in cutaneous wound healing.^156,157^ For more details regarding fibromodulin structure and function, please refer to recent thematic reviews.^16,47,115–117,119,121,158–162^

### Fibromodulin Exerts Pro- and Anti-inflammatory Effects by Binding to Complement and Complement Inhibitors

An increasing number of studies have demonstrated that fibromodulin plays a critical role in inflammatory diseases of the joint and influences the inflammatory response in wound healing, atherosclerosis, and heart failure.^163–165^ However, the mechanisms of this regulation are not fully clarified.

**Figure 4.**
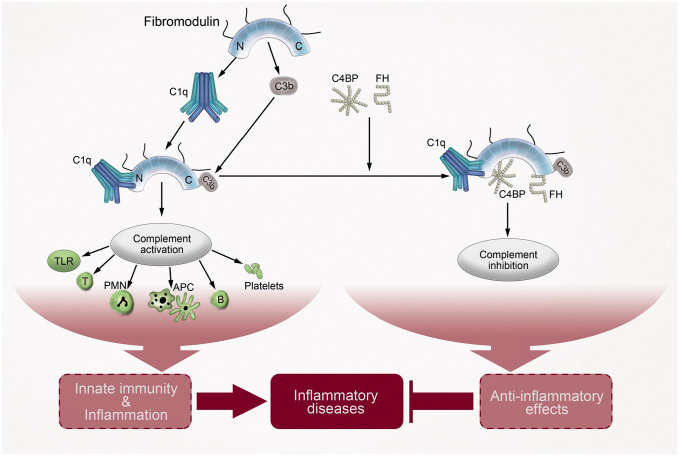
Fibromodulin modulates innate immune response and inflammation by both complement activation and complement inhibition. Fibromodulin, via its N-terminal site, binds with the complement element C1q, which results in the deposition of C3b, and together they initiate complement activation. An inflammatory signaling cascade is triggered, which includes TLR crosstalk, APC regulation, as well as PMN, B-cell, T-cell, and platelet activation, which contributes to innate immunity and inflammation. Overactivated and unresolved inflammation leads to inflammatory and autoimmune diseases. Contrarily, the binding of C4BP and FH to the fibromodulin/C1q/C3b complex leads to complement inhibition and therefore anti-inflammatory effects. Abbreviations: APC, antigen-presenting cells; C1q, complement 1q; C3b, complement 3b; C4BP, complement 4 binding protein; FH, factor H; TLR, Toll-like receptor; PMN, polymorphonuclear.

Several studies investigating joint diseases, for example, RA and osteoarthritis, strongly implicate that fibromodulin activates the classical and alternative pathways of complement via direct binding to complement elements C1q and C3b (Fig. 4).^163^ C1q is a multiprotein complex critically involved in the activation of the classical complement pathway.^166^ In contrast, C3b, formed by the cleavage of complement component 3, is a major trigger of alternative complement pathway.^167^ It is well established that fibromodulin interacts with the globular heads of C1q triggering the classical complement pathway, which subsequently leads to the deposition of C3b and activation of alternative complement pathway (Fig. 4).^165^ The activated complement system may further contribute to adaptive and cellular immune responses through crosstalk with TLRs,^168^ regulation of antigen-presenting cells,^169^ and activation of adaptive immune cells including PMNs,^170^ B- and T-lymphocytes,^171,172^ and platelets (Fig. 4).^173^ Thus, fibromodulin, via binding to the complement elements C1q and C3b, triggers a plethora of immune responses.

On the contrary, fibromodulin also interacts with the complement factor H (FH) and C4b-binding protein (C4BP), inhibitors of the complement system, limiting complement activation to the early part of the classical pathway (Fig. 4).^144,174,175^ It is of note that the binding sites on fibromodulin for C1q and FH do not overlap. The binding site for FH is localized at a position partially masked by the keratan sulfate chains, whereas C1q interacts with the N-terminal 10-kDa part of fibromodulin.^164^ Based on these mechanisms, it can be concluded that fibromodulin exerts anti-inflammatory effects.

Thus, it is conceivable that, under physiological conditions, fibromodulin, similar to biglycan,^50^ maintains a balance between pro- and anti-inflammatory responses. However, under disease conditions, this fine-tuning is disturbed and fibromodulin triggers sustained inflammation of tissues, for example, in joints.^176^

Even though there is no direct evidence that the soluble form of fibromodulin regulates the inflammatory response, there are some implications promoting this hypothesis. It is well known that in inflammatory joint diseases, the cartilage is degraded and fibromodulin is released into the synovial fluid.^177^ Furthermore, various fragments of fibromodulin bind with high affinity to either C1q or the complement inhibitors.^165^ Thus, it appears that soluble fibromodulin and its fragments are involved in complement-mediated regulation of inflammation.

Similar to fibromodulin, decorin and biglycan are also known regulators of the complement pathway.^144,163,174,175^ However, in contrast to fibromodulin, decorin and biglycan bind to the stalk of C1q, thereby inhibiting complement activity.^164^ Thus, SLRPs, through interactions with various complement factors, either activate or inhibit complement and tightly regulate the inflammatory response in a molecule-specific way.

### Fibromodulin Modulates TGF-β1 Activity in Inflammatory Diseases

Besides regulating the inflammatory response in joint disease, fibromodulin is also involved in the inflammatory process of cutaneous wound healing.^178^ Studies on fetal and adult rodent wound models provided evidence that elevated fibromodulin levels correlate with decreased TGF-β1 activity.^179^ This is based on the ability of fibromodulin protein core to sequester TGF-β1 in the ECM.^36,180^ In agreement, mice lacking fibromodulin displayed abnormal wound healing, which correlates with elevated inflammatory cell infiltration and accelerated epithelial cell migration. This was accompanied by increased type I TGF-β receptor levels in individual inflammatory cells at wound sites.^178,181^ Similar effects can be achieved by reducing fibromodulin abundance. Proteolytic degradation of fibromodulin by MMP2, MMP8, MMP9, MMP13, a disintegrin and metalloproteinase with thrombospondin motifs (ADAMTS)-4, and ADAMTS-5, decreased its abundance.^182,183^ For example, degradation of fibromodulin by MMP8 prevented fibromodulin–TGF-β complex formation, thereby increasing TGF-β bioavailability and M2-macrophage polarization.^184^

Thus, fibromodulin by sequestering TGF-β1 in the ECM prevents inflammation during would healing. Similar mechanisms were also described for decorin and biglycan.^155^ Based on differential localization of SLRPs in tissues,^185^ it appears that this is a common mechanism by which SLRPs protect various organ parts from excess of active TGF-β1.

### Involvement of Fibromodulin in Inflammatory and Autoimmune Diseases

Based on various mechanisms of fibromodulin-mediated regulation of inflammation described above, a broad spectrum of diseases is expected to be influenced by this proteoglycan. However, the number of publications describing the role of fibromodulin in inflammatory and autoimmune diseases is still limited.

There is evidence that renal fibromodulin is markedly overexpressed and accumulated in patients suffering from membranous glomerulonephritis and diabetic nephropathy.^108,185^ Furthermore, enhanced abundance of cardiac fibromodulin was reported in human and animal model of heart failure.^152,186,187^ However, mice deficient in fibromodulin challenged by pressure overload displayed only mildly exacerbated hypertrophic remodeling associated with attenuated cardiac immune cell infiltration.^152^ Additional support for the involvement of fibromodulin in inflammatory diseases is provided by reports addressing its role in atherosclerosis.^188,189^ Higher fibromodulin content along with enhanced levels of inflammatory cytokines was detected in atherosclerotic plaques from patients with diabetes mellitus.^189^ In agreement, lack of fibromodulin in apolipoprotein E–deficient mice leads to decreased vascular lipid retention and reduced plague development.^188^ Furthermore, numerous studies indicate enhancement of fibromodulin in the articular cartilage under inflammatory conditions.^160,176,190^

## Future Perspectives

It is fascinating that SLRPs, despite their structural and functional similarities, modulate innate immune and inflammatory responses in a molecule-specific manner. Although certain receptors, mediators, and signaling pathways, such as TLRs, TGFβ, and NF-κB, respectively, obviously overlap between one or more SLRPs, it is becoming increasingly clear that SLRPs select unique receptors, coreceptors, adaptor molecules, and mediators to achieve a specific cellular outcome. For example, the same SLRP can start molecular pathways triggering the release of pro-inflammatory cytokines or inhibiting them. This is achieved by either promoting or impeding the pro-inflammatory signaling mechanisms. This selection also appears to be regulated at the tissue level, as the presence of the same SLRPs, as in the case of decorin, worsens the disease phenotype in pSS but has protective effects on IBD, and this regulation is particularly important from a therapeutic point of view.

Among the 18 distinct gene products belonging to the family of SLRPs, signaling mechanisms and functional relevance of biglycan, decorin, lumican, and fibromodulin are the best characterized. Although all four SLRPs, in their soluble form, act as signaling molecules to regulate inflammation, many signaling pathways are still not completely understood. Further breakthrough in our understanding of the functional role of the proteoglycans in physiological and diseased states can be achieved by additional mechanistic studies focused on different cell lines, in vivo models, and collected patient data. For example, based on our current knowledge, we know that biglycan and decorin act as canonical ECM-derived DAMPs, and lumican appears to behave as an accessory molecule that presents pathogens to the innate immunity receptors. Additional evidence for the role of lumican as a helper molecule, and not a direct trigger, in inflammatory reactions is further provided by its role in promoting PMN migration and extravasation. An intriguing question therefore arises: Is lumican also involved in presenting ECM-DAMPs to TLRs? Identification of such novel interactions can have significant biological relevance. Similarly, the involvement of fibromodulin as part of the inflammatory response pathway is undoubted, yet mechanistic insides of these processes are not well characterized.

Growing numbers of reports demonstrate that SLRPs modulate both pro- and anti-inflammations. Even canonical DAMPs like biglycan and decorin exert anti-inflammatory effects. A common mechanism by which SLRPs inhibit inflammation is by their ability to regulate autophagy. Thus, it would be interesting to clarify whether decorin, similar to biglycan, also promotes a similar switch between inflammation and autophagy by choosing specific coreceptors of TLR4. Studies that investigate the roles of SLRP in mediating receptor crosstalk to initiate either inflammation or selective autophagy would therefore be of high interest, especially as it sheds light on our understanding of the molecular pathogenesis of inflammatory and autoimmune diseases.

Besides their regulatory role in innate immunity, all four SLRPs also play distinct roles in shaping the adaptive immune response. The contrary effects of biglycan and lumican on Th17 cells further highlight the molecule-specific role of SLRPs in immune reactions. Much is definitely still not known regarding SLRP-mediated signaling, and further research is warranted. Studies that will investigate different SLRPs in the same cellular and tissue context would provide more definitive answers to augment our overall understanding of SLRPs. Nevertheless, existing data demonstrate the complex interplay between cellular mediators and the tight regulation of molecular pathways observed in SLRP-mediated signaling. The ultimate query that needs to be answered is whether the biological role of SLRPs is to initiate or resolve inflammation, and such biological question provide a promising outlook for future studies.
